# Advice to Young Dentists

**Published:** 1873-10

**Authors:** 


### ADVICE TO YOUNG DENTISTS.

BY C. JR.

*Where shall I locate?* This is the first question most young graduates put to themselves, and, strange as it may appear, most of them decide upon starting away from the location where they have been brought up. This question has been asked the writer frequently, and for the benefit of all concerned, I will place upon paper my views of the subject, and why; and how to succeed after starting an office.

First, young man, open an office where you are somewhat acquainted, but do no work for an advertisement, or gratis; no matter for whom you operate, charge them for your labor, and if for a friend, charge well. Ask no favors and give none in your business, is a very good motto, and should be lived up to. If you do your work well for your acquaintances, and they are really your *friends*, they will not object to paying for it, and the more they pay, the more pleased they will be to show the work, and to say who did it. Here you will have a good influence at work, and it will not be at your expense.

Whereever you start, under all circumstances, act as becomes a gentleman. Do not dissipate; go into good society, but do not carry your business with you into the parlor; do not advertise *yourself*; do not boast of your own operations; let others speak for you; and you will, if you are polite, soon find your society sought, not only in the parlor, but in the office. Never speak of a brother dentist as a quack, even if he be one; for this shows a bit of envy. Therefore, start where you are acquainted, as it will be much easier to follow the above advice.

Now for those who desire to start away from home, for reasons best known to themselves: I say by all means open your office, where you will have lively competition; and that will be in a city. You will ask why? Here it will be more difficult for you to get into society, and you will be compelled to form your acquaintances. If you act the gentleman in your office, your patients (for you will soon have at least one, if only to have a tooth extracted,) will soon discover that you are polite, obliging and careful, (and do not show your politeness to those only who dress well, as I am sorry to say many dentists do, when they show it at all,) and will not only call again, but will soon herald you, as a gentleman and dentist. And do work for *charity* rather than for a *small* fee. Now you may say, "I will starve before I can build up a practice." I will answer by saying, Nonsense. I have never heard of a single instance of a dentist starving, or of one becoming poorer, who charged good prices for his labor ; but I have heard of many who have given up, because they did not charge enough to make their living ; and in a large city, the demand is greater for good operators, than in the country, and then operations are appreciated better.

The way to succeed after opening your office is very simple. As before said, politeness and gentlemanly conduct are the first requisites, after obtaining your outfit, and after having procured a suitable location for your office. The next thing, when your patient arrives, unless it is a case requiring immediate attention, make an appointment with him for a few days hence, having him to infer that you are doing some business ; a little deception in this way is not improper. After thus far proceeding, when your patient comes to fulfill the engagement, proceed to work, being gentle in handling, but above all else do your work skillfully. If it is a case requiring treatment, and you do not think yourself competent to treat properly, call in some more experienced operator for advice. Do not fear your practice will be taken away from you on that account ; such occurrences rarely occur ; but should such present itself, remember it is better for your reputation, that you lose a patient rather than injure him, and yourself, perhaps, forever. This fact cannot be too strongly impressed upon the minds of young dentists.

Make no flaming display of signs ; don't advertise in the newspapers unless it is simply your card ; and do not be discouraged. Do what you have to do well : charge well for your labor, for remember you can not operate for many years, and while you are able you must make a competency. Treat your brother dentist as a professional equal ; obtain his good will even if it is worth nothing; command his respect. Never cease studying; spend all your spare moments, after taking necessary exercise, in reading dental works; read all the dental publications, so that you will be up with the times. Observe the work of others at every opportunity; attend conventions, and society meetings; and you will soon find that your business is enlarging, your reputation progressing, and your practice increasing.

